# Cancer stem cells in personalized therapy: mechanisms, microenvironment crosstalk, and therapeutic vulnerabilities

**DOI:** 10.3389/fcell.2025.1619597

**Published:** 2025-07-30

**Authors:** Ling Yin, Shoubing Zhou, Hongliang Zhang, Yuhua Shang, Songquan Wu, Tengchuan Jin

**Affiliations:** ^1^ Center of Disease Immunity and Intervention, College of Medicine, Lishui University, Lishui, China; ^2^ College of Medicine, University of Florida, Gainesville, FL, United States; ^3^ Division of Infectious Diseases and Geographic Medicine, Department of Internal Medicine, University of Texas Southwestern Medical Center, Dallas, TX, United States; ^4^ Institute of Health and Medicine, Hefei Comprehensive National Science Center, Hefei, Anhui, China; ^5^ Key Laboratory of Anhui Province for Emerging and Reemerging Infectious Diseases, Hefei, China; ^6^ Division of Life Sciences and Medicine, Laboratory of Structural Immunology, Key Laboratory of Immune Response and Immunotherapy, University of Science and Technology of China, Hefei, China; ^7^ Anhui Genebiol Biotech. Ltd., Hefei, China; ^8^ Biomedical Sciences and Health Laboratory of Anhui Province, University of Science and Technology of China, Hefei, China; ^9^ Clinical Research Hospital of Chinese Academy of Sciences (Hefei), University of Science and Technology of China, Hefei, China; ^10^ Division of Life Sciences and Medicine, Department of Infectious Diseases, The First Affiliated Hospital of USTC, University of Science and Technology of China, Hefei, Anhui, China

**Keywords:** cancer stem cells, metabolic reprogramming, immune evasion, organoids, personalized therapy

## Abstract

Cancer stem cells (CSCs) drive tumor progression, therapy resistance, and metastasis through unique membrane biology, glycosylation patterns, and metabolic adaptations. CSCs exhibit a distinct glycocalyx profile enriched in hyaluronan, heparan sulfate, and sialylated glycans, facilitating immune evasion, adhesion, and survival. Key signaling pathways—Wnt/β-catenin, Hedgehog, Notch, JAK/STAT, TGF/SMAD, and PI3K/AKT/mTOR—regulate CSC stemness and therapeutic resistance. Emerging biomarkers (CD44, CD133, ALDH1, EpCAM) and targeted therapies (CAR-T cells, miRNA modulation, lipid metabolism inhibitors) show promise in disrupting CSC resilience. Advances in single-cell omics, CRISPR screening, and patient-derived organoids (PDOs) enhance CSC characterization and precision medicine applications. However, challenges remain in standardizing organoid cultures, replicating tumor microenvironments, and overcoming CSC plasticity. Integrating CSC-targeted strategies with conventional therapies may improve clinical outcomes by eradicating therapy-resistant populations and preventing relapse. This review underscores the need for innovative combination therapies to eradicate CSCs and improve clinical outcomes, while addressing challenges in biomarker validation, therapeutic resistance, and translational applications.

## Introduction

Cancer, “a wound that never heals”, is a leading cause of death worldwide, which is characterized by the uncontrolled proliferation of abnormal cells and aberrant recognition of immune system (Ushijima et al., 2021; Hanahan, 2022). The global World Health Organization (WHO) survey on universal health coverage (UHC) and cancer show that only 39% of participating countries covered the basics of cancer management as part of their financed core health services for all citizens, while only 28% of participating countries additionally covered care for people who require palliative care (WHO, 2024).

Cancer stem cells (CSCs) represent a subpopulation of tumor cells with self-renewal capacity, differentiation potential, and resistance to conventional therapies, driving tumor initiation, progression, metastasis, and recurrence (Kreso and Dick, 2014; Clarke, 2019). Unlike differentiated cancer cells, CSCs exhibit unique membrane biology and glycosylation patterns that contribute to their malignant behavior. A distinctive glycocalyx profile, characterized by the overexpression of specific glycans, proteoglycans, and glycosylation enzymes, plays a pivotal role in CSC interactions with the tumor microenvironment (TME), immune evasion, and adhesion-mediated metastasis (Kang et al., 2018; Zheng et al., 2022). Notably, glycosaminoglycans (GAGs) such as hyaluronan and heparan sulfate facilitate pro-survival signaling (e.g., CD44-HA and Wnt/β-catenin), while proteoglycans like syndecan-1 and glypican-3 enhance epithelial-mesenchymal transition (EMT) and growth factor signaling (Wang et al., 2025; Teixeira et al., 2020). Furthermore, CSC glycocalyx components recruit immunosuppressive cells (e.g., Tregs, MDSCs, TAMs) and engage immune checkpoints (e.g., PD-L1, Siglec receptors), promoting an immune-privileged niche (Ju et al., 2022; Oshimori, 2020).

Beyond membrane dynamics, CSCs rely on exosome-mediated crosstalk with the TME to foster angiogenesis, stromal activation, and drug resistance (da Costa et al., 2021). CSC-derived exosomes transfer EMT-inducing factors (e.g., Twist, Snail, TGF-β) and oncogenic miRNAs, reinforcing stemness and metastatic potential (Babaei et al., 2021). Metabolic adaptations, particularly in lipid metabolism (e.g., FAO, lipogenesis), further sustain CSC resilience under stress. Key pathways such as Wnt/β-catenin, Hedgehog, Notch, JAK/STAT3, and PI3K/AKT/mTOR orchestrate CSC maintenance (Su et al., 2015; Wang et al., 2012; Sari et al., 2018; Wang T. et al., 2018; Karami et al., 2022), while biomarkers like CD44, CD133, and ALDH1 aid in their identification and targeting (Martinez-Gamero et al., 2021; Azzoni et al., 2022). Recently, three-dimensional (3D) organoids have bridged the gap between *in vitro* two-dimensional (2D) cell lines and *in vivo* mouse models for cancer research, which are established from adult stem cells (ASCs) and pluripotent stem cells (PSCs) (Sato et al., 2009; McCauley and Wells, 2017).

Emerging technologies—single-cell omics, CRISPR screens, and AI—are refining CSC characterization and therapeutic strategies. Patient-derived organoids (PDOs) now enable precision medicine by modeling tumor heterogeneity and therapy responses. However, clinical translation faces challenges, including CSC plasticity, niche-mediated resistance, and toxicity concerns. This review synthesizes advances in CSC biology, therapeutic targeting, and innovative models, offering insights into overcoming CSC-driven therapy resistance and improving cancer outcomes.

## Methodological overview of CSC identification and validation

To provide a robust foundation for our discussion of cancer stem cells (CSCs) and their role in personalized therapy, it is essential to first establish the experimental framework used to identify and validate these elusive cell populations. The characterization of CSCs relies on a combination of surface marker analysis, functional assays, and *in vivo* validation, each contributing unique insights into their biological properties.

Surface marker-based isolation represents one of the most widely adopted approaches, with markers such as CD44, CD133, and ALDH1 serving as key indicators of CSC populations (Rugg-Gunn, 2022). Flow cytometry enables the precise enrichment of these subpopulations, with specific combinations—such as CD44^+^CD24−/low cells in breast cancer—providing clinically relevant signatures (Chen et al., 2020). Complementing this, the Aldefluor assay detects elevated aldehyde dehydrogenase (ALDH) activity, an enzyme frequently overexpressed in CSCs, allowing for fluorescence-based separation of ALDH-high cells (Duan et al., 2023; Del Vecchio et al., 2024).

Functional assays further validate CSC properties, with sphere formation being a hallmark of self-renewal capacity (Bahmad et al., 2018; Perry and Li, 2010). When cultured in serum-free, non-adherent conditions, CSCs generate three-dimensional spheres, reflecting their ability to proliferate and maintain stemness over multiple passages. This assay is particularly valuable for assessing the hierarchical organization of tumors (Ma et al., 2019).

The gold standard for CSC validation remains *in vivo* tumorigenicity assays, wherein sorted cells are injected into immunocompromised mice to evaluate their tumor-initiating potential (Skidan and Steiniger, 2014; Sato et al., 2019). Notably, even a minimal cell population can suffice to generate tumors, underscoring the profound biological potency of CSCs. This approach not only confirms stemness but also provides critical insights into therapeutic resistance mechanisms (Makena et al., 2020).

Collectively, these methodologies establish a framework for identifying and characterizing CSCs, bridging molecular observations with clinically relevant phenotypes. By integrating these techniques, researchers can better delineate the molecular pathways driving CSC maintenance and develop targeted therapies to disrupt their survival and proliferation.

### Membrane biology and glycosylation of CSCs

The unique membrane biology and glycosylation patterns of CSCs play a pivotal role in their interactions with the TME and their resistance to therapies, which is essential for developing targeted therapeutic strategies. CSCs possess a unique glycocalyx profile characterized by the overexpression of specific glycans, proteoglycans, and glycosylation enzymes, which serve as critical mediators of their malignant behavior (Park et al., 2024). CSC glycocalyx enriches in certain glycosaminoglycans (GAGs), such as hyaluronan and heparan sulfate, which facilitate interactions with the tumor microenvironment and activate pro-survival signaling pathways. CSC glycocalyx also displays an upregulation of proteoglycans like syndecan-1 and glypican-3, which modulate growth factor signaling and enhance epithelial-mesenchymal transition (Wei et al., 2024).

Glycocalyx components, particularly hyaluronan and chondroitin sulfate proteoglycans, create a pro-tumorigenic microenvironment by recruiting immunosuppressive cells such as regulatory T cells (Tregs), myeloid-derived suppressor cells (MDSCs), and tumor-associated macrophages (TAMs), which further inhibit effector immune responses and foster CSC survival. Glycocalyx components, such as mucins and sialylated glycans, interact with inhibitory immune checkpoints like PD-L1 and Siglec receptors (Buffone and Weaver, 2020). Overexpression of sialic acid residues (e.g., polysialic acid) engages Siglecs on immune cells, transmitting “do not eat me” signals that suppress phagocytosis by macrophages and dendritic cells. Similarly, mucins can directly bind to immune receptors, dampening cytotoxic responses (Ayyalasomayajula and Cudic, 2024). Consequently, targeting glycocalyx components represents a promising therapeutic strategy to overcome CSC-mediated immune escape and improve cancer immunotherapy efficacy.

The glycocalyx facilitates CSC adhesion through specific ligand-receptor binding, such as CD44-Hyaluronan (HA) interaction, selectin-mediated adhesion, and integrin activation (Kanyo et al., 2020). The glycocalyx acts as a physical barrier and shock absorber, allowing CSCs to withstand hemodynamic shear forces during circulation, which facilitates transient adhesion and firm attachment to vascular endothelia (Lipowsky, 2018). Enzymatic modifications alter glycocalyx density, which affect adhesion strength and migratory capacity. The strategies for disrupting glycocalyx-mediated adhesion include enzymatic degradation of glycocalyx components (e.g., hyaluronidase), inhibition of glycan-binding receptors (e.g., anti-CD44 antibodies, selectin antagonists), nanotechnology-based approaches (nanoparticles).

CSCs exhibit distinct glycosylation patterns compared to differentiated cancer cells and normal stem cells, which include truncated O-Glycans (e.g., Tn and Sialyl-Tn antigens), sialylation (e.g., α2,6- and α2,3-linked sialic acids), fucosylation (e.g., Lewis antigens, Core fucosylation), heparan sulfate proteoglycans (HSPGs), N-Glycan branching (e.g., β1,6-GlcNAc branching by MGAT5) (Rømer et al., 2021; Mun and kley, 2022; Shan et al., 2019; Onyeisi et al., 2020; Taniguchi et al., 2022). Glycan-based biomarkers hold great promise for CSC detection and therapeutic targeting, which include CD44 variants (CD44v) with unique glycosylation, CD133 (Prominin-1) glycosylation, podocalyxin (PODXL), integrins with altered glycosylation, and glycosphingolipids (GSLs) (Hu et al., 2019).

Understanding the unique glycocalyx profile of CSCs provides a foundation for exploring their interactions with the TME and their role in therapy resistance, which leads us to examine the mechanisms by which CSCs communicate with their surroundings and maintain their stemness, including exosome-mediated crosstalk and EMT.

### Exosomes, EMT, and CSC crosstalk

Exosomes, a subset of extracellular vesicles (EVs) ranging from 30 to 150 nm in diameter, mediate bidirectional crosstalk between CSC and TME, which influence tumor initiation, progression, metastasis, therapeutic resistance, and immune evasion (Li et al., 2024). CSC-derived exosomes can reprogram neighboring non-CSC tumor cells into a stem-like phenotype, promoting tumor heterogeneity. CSC-secreted exosomes contribute to the formation of a supportive niche by inducing angiogenesis, activating fibroblasts, and remodeling the extracellular matrix (Zabeti et al., 2024). CSC-secreted exosomes can inhibit T-cell and NK-cell activity while promoting regulatory T-cell (Treg) expansion, which establish an immune-privileged niche conducive to CSC survival (Yang and Teng, 2023).

CSC-secreted exosomes contribute to metastatic dissemination by priming pre-metastatic niches and enhancing the invasiveness of cancer cells, which transfer pro-metastatic factors to neighboring stromal and tumor cells, promoting angiogenesis, extracellular matrix (ECM) remodeling, and immune evasion (Li X. et al., 2022; Rashid et al., 2023). CSC-exosomes confer chemoresistance by transferring drug-efflux pumps, anti-apoptotic proteins, and resistance-associated miRNAs to sensitive cancer cells, which modulate the TME by inducing a cancer-associated fibroblast phenotype to secrete protective cytokines and shield CSCs from therapy (Chung et al., 2021; Zhuang et al., 2023).

Epithelial-mesenchymal transition (EMT), a critical process in cancer metastasis and recurrence, could involve the transformation of epithelial cells into a mesenchymal phenotype, which enhances migratory capacity, invasiveness, and resistance to apoptosis (Mortezaee et al., 2022). EMT transcription factors serve as molecular bridges between cellular plasticity and stemness, which enable CSCs to evade therapy and drive metastasis (Lu and Kang, 2019). CSC-derived exosomes carry a repertoire of EMT-promoting molecules, including transcription factors (e.g., Twist, Snail, Slug, Zeb1/2), miRNAs (e.g., miR-21, miR-10b, miR-155), and cytokines (e.g., TGF-β, IL-6), which modulate signaling pathways such as Wnt/β-catenin, NF-κB, and PI3K/Akt, leading to the downregulation of epithelial markers (E-cadherin) and upregulation of mesenchymal markers (N-cadherin, vimentin, fibronectin) (Debnath et al., 2022; Lin et al., 2014; Ray et al., 2023; Kitazawa et al., 2024; Guo et al., 2024).

EMT-induced stemness confers tumor cells with increased self-renewal capacity, plasticity, and adaptability, contributing to tumor heterogeneity and therapy evasion, which could promote resistance to conventional therapies (chemotherapy, radiotherapy, and targeted therapies) through multiple mechanisms, such as enhanced DNA repair and survival pathways, drug efflux pumps, metabolic adaptations, and immune evasion (Lei et al., 2024; Roy et al., 2021; Voon et al., 2013). Given the role of EMT-induced stemness in therapeutic resistance, novel strategies are needed to target this aggressive cell population, which include epigenetic modulators (reverse EMT-associated transcriptional reprogramming), immune-based strategies (CSC-targeted vaccines or CAR-T cells engineered to recognize EMT/CSC markers), combination therapies (target both EMT and stemness pathways) (Peixoto et al., 2019; Qian et al., 2020; Sadrkhanloo et al., 2022).

The role of exosomes and EMT in CSC biology highlights the importance of understanding the metabolic adaptations that sustain CSC resilience. The metabolic adaptations of CSCs, particularly in lipid metabolism, are crucial for their survival and stemness maintenance. These metabolic pathways provide energy and biosynthetic precursors, enabling CSCs to thrive in challenging microenvironments.

### Lipid metabolism in CSCs

Lipid metabolism, including fatty acid oxidation (FAO) and *de novo* lipogenesis, could not only fulfill the bioenergetic and biosynthetic demands of CSCs but also regulate redox homeostasis, signaling pathways, and epigenetic modifications, thereby maintaining stemness and survival under stress conditions (Singh et al., 2024). CSCs exhibit heightened reliance on mitochondrial FAO to generate adenosine triphosphate (ATP), especially in nutrient-deprived or hypoxic microenvironments, which is mediated by upregulation of key enzymes such as carnitine palmitoyltransferase 1 (CPT1) and is linked to chemoresistance via enhanced oxidative stress mitigation (Mascaraque et al., 2024). Lipogenesis, *de novo* fatty acid synthesis, driven by acetyl-CoA carboxylase (ACC) and fatty acid synthase (FASN), supports CSC membrane biogenesis, lipid raft formation, and oncogenic signaling (Liu Z. et al., 2023). Targeting lipid metabolism in CSCs presents a promising strategy to overcome therapy resistance, which include FAO inhibitors (e.g., etomoxir, ranolazine) and lipogenesis inhibitors (e.g., orlistat, TVB-3166) (Ahn et al., 2024; Batchuluun et al., 2022).

Key enzymes in lipid metabolism, such as carnitine palmitoyltransferase 1A (CPT1A) and ATP-citrate lyase (ACLY), have been implicated in CSC maintenance. CPT1A, the rate-limiting enzyme in mitochondrial FAO, facilitates the transport of long-chain fatty acids for β-oxidation as well as supports energy production under metabolic stress (e.g., hypoxia or nutrient deprivation) (Zhu et al., 2025). Due to the upregulation of CPTIA in CSCs promoting chemoresistance and metastasis, CPT1A inhibitors (e.g., etomoxir or novel analogs) are promising candidates to disrupt CSC resilience (Zha et al., 2025). ACLY, a central enzyme linking glycolysis to lipogenesis, converts citrate to acetyl-CoA for fatty acid synthesis, fueling membrane production and oncogenic signaling (e.g., via histone acetylation) (Bort et al., 2020). Due to the overexpression of ACLY in CSCs correlating with poor prognosis, ACLY inhibitors (e.g., SB-204990 or BMS-303141) show efficacy in suppressing CSC populations, tumor growth, and metastasis (Sola-García et al., 2023; Puertas-Umbert et al., 2025).

### Pathways and cancer immunotherapy

**FIGURE 1:**
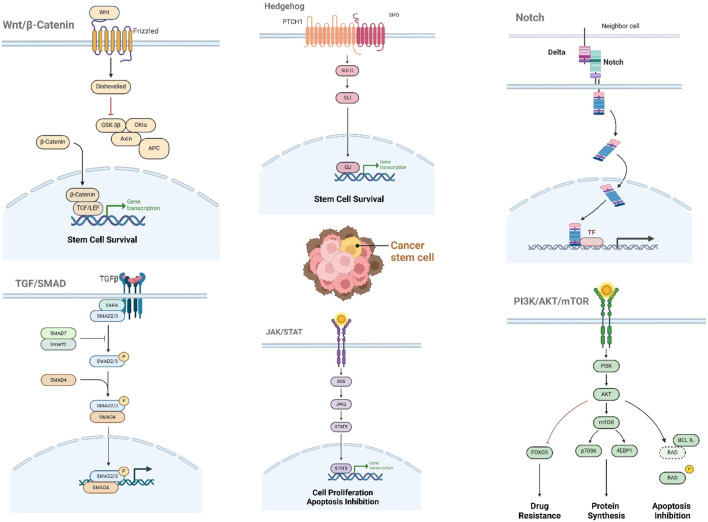
Core signaling pathways regulating cancer stem cell (CSC) properties. Illustrated pathways (WNT/β-catenin, Hedgehog, Notch, JAK-STAT, TGF-β/SMAD, PI3K/AKT/mTOR) govern CSC self-renewal, survival, metastasis, and therapy resistance. Each pathway modulates: Proliferation (WNT/β-catenin, Hedgehog, Notch), Survival/Apoptosis evasion (PI3K/AKT/mTOR, JAK-STAT), Metastasis (TGF-β/SMAD, JAK-STAT), Stemness maintenance (Cross-talk between all pathways).

This section focuses on the signaling pathways that regulate CSC maintenance and survival, highlighting potential therapeutic targets. We will discuss emerging strategies for disrupting these pathways to improve treatment outcomes. Many highly complex and evolutionarily conserved signaling pathways are interwoven networks of signaling mediators to regulate CSC growth, which also induces the expression of downstream genes (apoptosis, anti-apoptotic, proliferation, and metastasis) (Zeng et al., 2023). At present, the main signaling pathways are the WNT/β-Catenin, hedgehog, Notch, JAK/STAT, TGF/SMAD, and PI3K/AKT/mTOR signaling pathways (Figure 1).

The WNT/β-Catenin signaling pathway in CSCs, consisting of 19 Wnt ligands and more than 15 receptors, could regulate stemness, tumorigenesis, metastasis, apoptosis, and differentiation across diverse cancer types, including lung, liver, thyroid, colorectal, cervical, and glioblastoma (Liu et al., 2021; Feng et al., 2021; He et al., 2021). The hedgehog signaling pathway in CSCs, consisting of extracellular Hh ligands, transmembrane protein PTCH and SMO, intermediate transduction molecules, as well as downstream molecule GLI, plays a pivotal role in maintaining stemness, promoting initiation, and enhancing tumorigenicity in liver cancer, bladder cancer, breast cancer, and glioblastoma (Mok et al., 2022; Li et al., 2016; Qin et al., 2018). The Notch signaling pathway in CSCs, consisting of the Notch receptor, Notch ligand, CBF-1, hairless suppressor, Lag (CSL), DNA binding protein, and downstream target genes, is associated with metastasis, stemness maintenance, tumorigenesis, differentiation, and immune regulation of CSCs in various tumors such as glioma, breast cancer, renal cancer, and ovarian cancer (Chen et al., 2018; Rajakulendran et al., 2019; Cao et al., 2023). The JAK/STAT signaling pathway in CSCs, consisting of tyrosine kinase-related receptors that receive signals, tyrosine kinase JAK that transmits signals, and transcription factors STAT, is intricately linked to stemness, tumorigenesis, metastasis, and metabolic reprogramming of CSCs in glioblastoma, osteosarcoma, liposarcoma, liver cancer, prostate cancer, gastric cancer, thyroid cancer, and breast cancer (Garner et al., 2019; Xiong et al., 2024; Shiraiwa et al., 2019). The TGF/SMAD signaling pathway in CSCs, consisting of two ligand groups (TGF-β/activin and BMP/GDF), plays a pivotal role in metastasis, tumorigenesis, and stemness of glioma, breast cancer, prostate cancer, pancreatic cancer, and oral/esophageal squamous cell carcinoma (Zhao et al., 2019; Nong et al., 2022; You et al., 2020). The PI3K/AKT/mTOR signaling pathway in CSCs, consisting of intracellular phosphatidylinositol kinase PI3K, serine/threonine kinase AKT, downstream target mTOR, and negative regulator PTEN, is intricately linked to metastasis, stemness, and tumorigenicity in gastric cancer, endometrial cancer, ovarian cancer, lung cancer, osteosarcoma, as well as head and neck squamous cell carcinoma (Wang et al., 2021; Li et al., 2021; Wang JH. et al., 2020).

The intricate crosstalk among multiple signaling pathways regulates the tumorigenicity, differentiation, and metastasis capabilities of CSCs. WNT/β-Catenin, hedgehog, Notch, and TGF-β pathways influence the differentiation of colorectal CSCs (Regan et al., 2017). The interaction between IL-6/JAK/STAT3 and TGF-β/Smad signaling induces the proliferation and metastasis of lung CSCs (Liu et al., 2014). TGF-β1 activating induces lncRNA NKILA expression to block NF-κB signaling in breast CSCs (Wu et al., 2018). PI3K/AKT/mTOR signaling upregulates STAT3 expression to promote the survival and proliferation of breast CSCs (Zhou et al., 2007). The collaboration of PI3K/AKT/mTOR with Sonic Hedgehog pathways could inhibit the growth of pancreatic CSCs (Zuo et al., 2015).

The signaling pathways regulating the maintenance and survival of CSCs have become oncology targets, with early clinical trials for Notch and hedgehog pathway inhibitors. There are three major clinical methods for inhibiting Notch signaling, such as secretase inhibition (γ-secretase inhibitor, GSI), Notch receptor or ligand antibodies, and combination therapy. There are several GSIs entering the clinical trial stage, such as MK-0752 (NCT00100152), RO4929097 (NCT01154452), Nirogacestat/PF-03084014 (NCT01981551), BMS-906024 (NCT01292655), BMS-986115 (NCT01986218), CB-103 (NCT03422679), Crenigacestat/LY3039478 (NCT02836600), and LY900009 (NCT01158404) (Yang L. et al., 2020). The hedgehog signaling pathway regulates target gene expression through smoothened (SMO)-mediated nuclear transfer of transcription factors. Three oral SMO antagonists, Vismodegib (GDC-0449), Sonidegib (LDE225), and Glasdegib (PF-04449913), have been approved by the Food and Drug Administration (FDA), which show significant activity in locally advanced and metastatic basal cell carcinoma, as well as in acute myeloid leukemia (Sekulic et al., 2012; Dummer et al., 2016; Norsworthy et al., 2019).

### Biomarkers and therapeutic approaches

Cancer biomarkers are molecular, cellular, tissue, and process-based alterations providing indications of current and future cancer behaviors, which are applied in pharmaceutical discovery and preclinical development, clinical trials, and patient care (Hayes et al., 1996; Xiao et al., 2021). Cancer biomarkers could be clarified into two categories: clinical trials (chemoprevention, screening, diagnosis, prognosis, prediction, treatment stratification, therapy monitoring, posttreatment surveillance, risk stratification, and risk management) (Borrebaeck, 2017; Le-Rademacher et al., 2018; Gonzalez-Ericsson et al., 2020; Jordan and Thomas, 2023) and drug development (target validation, early compound screening, pharmacodynamic assays, patient selection, and surrogate endpoint) (Pors and Moreb, 2014; Twomey et al., 2017; Lara et al., 2020; Wang E. et al., 2024). The identification of CSC biomarkers has facilitated the development of targeted therapies aimed at disrupting CSC maintenance and survival. These biomarkers serve as diagnostic, prognostic, and therapeutic targets, providing insights into CSC biology and potential therapeutic vulnerabilities.

CSCs biomarkers, such as cell surface markers, signaling pathways, transcription factors, and drug transporters, are involved in self-renewal, immune evasion, tumor metastasis, tumor re-growth, tumor relapse, and therapy resistance (Yehya et al., 2023). CSC biomarkers could serve as diagnostic (resistance), therapeutic (metastasis and tumor stage/size/resistance), and prognostic (survival and resistance) approaches in multiple deadliest cancers, such as lung cancer, liver cancer, breast cancer, gastric cancer, prostate cancer, bladder cancer, and colon cancer (Table 1).

**TABLE 1:** Clinically validated cancer stem cell biomarkers in solid tumors: Functional mechanisms and diagnostic utility.

Biomarker	Primary clinical role	Key molecular function	Prostate cancer	Colon cancer	Bladder cancer	Breast cancer	Lung cancer	Liver cancer	Gastric cancer
CD24	Diagnostic (Resistance)	WNT/β-Catenin, MAPK, Notch regulation	Metastasis	Metastasis association	CSC niche maintenance	Therapy resistance		Prognostic marker	Chemoresistance
CD44	Therapeutic (Metastasis)	VEGFR/PI3K/Akt activation; c-Met coreceptor	Therapy resistance	Metastasis	Prognostic marker	Metastasis	Tumor initiation	Stemness maintenance	EMT induction
CD49f	Therapeutic (Niche)	Tumor-microenvironment crosstalk	Tumor initiation	CSC isolation marker	CSC niche maintenance	Self-renewal regulator	Stemness marker		
CD133	Diagnostic (Relapse)	WNT/Ras/Notch pathway modulation	Chemoresistance; Prognostic	Recurrence predictor	Recurrence predictor	Self-renewal regulator	Prognostic marker	Prognostic marker	Stemness marker
EpCAM	Therapeutic (CTC target)	Circulating tumor cell anchor; EMT regulator	Stemness maintenance	Wnt signaling activator	Tumorigenicity driver	Prognostic marker	Metastasis driver	Stemness maintenance	Tumorigenicity driver
CXCR4	Therapeutic (Metastasis)	WNT/NF-κB mediated metastasis	Metastasis driver	Metastasis driver		Metastasis driver	Metastasis driver		Prognostic marker
LGR5	Therapeutic (Self-renewal)	Wnt/β-catenin signaling receptor		Wnt signaling activator		Self-renewal regulator	Prognostic marker		Stemness maintenance
ALDH	Diagnostic (Resistance)	Tumor dedifferentiation/EMT driver	Chemoresistance; Prognostic	Chemoresistance marker	CSC activity marker	Chemoresistance marker	Prognostic marker		Prognostic marker
ABCG2	Therapeutic (Resistance)	Drug efflux pump	Drug efflux mediator	Chemoresistance marker	Drug efflux mediator	Chemoresistance marker	Drug efflux mediator		
EGFR	Therapeutic (Target)	Stemness/dormancy regulator	Therapy target	Metastasis driver		Stemness regulator	Survival regulator	Tumorigenicity driver	

CD24, also known as Heat Stable Antigen (HSA), functions as a cell-cell adhesion molecule to mediate WNT/β-Catenin, MAPK, PI3K/AKT/mTOR, Notch, and hedgehog pathways (Zhang et al., 2017a; Kapeleris et al., 2020; Ooki et al., 2018; Al-Hajj et al., 2003; Wang et al., 2018b; Zhang et al., 2011). CD44, also known as Homing Cell Adhesion Molecule (HCAM) and Phagocytic Glycoprotein-1 (Pgp-1), recruits ezrin/radixin/moesin (ERM) proteins to interact with VEGFR and to activate the PI3K/Akt and Src/MAPK pathways, which also serves as a c-Met co-receptor (Hurt et al., 2008; Du et al., 2008; Chan et al., 2009; Koh et al., 2021; Leung et al., 2010; Asai et al., 2019). CD24 has been investigated in combination with CD44 in breast cancer, prostate cancer, and gastric cancer (Zhang et al., 2011; Hurt et al., 2008; Koh et al., 2021). CD49f, also known as integrin α6, is the only conserved biomarker in more than 30 different stem cell populations, which mediates the stem cell niche through interactions with the extracellular matrix as well as communication between tumor cells and tumor microenvironment (Marcinkiewicz et al., 2012; Bo et al., 2009; Zhang et al., 2020; Zhang et al., 2017b). CD90, also known as THY1, is glycophosphatidylinositol anchored cell surface protein in T cell adhesion and signal transduction, which marks hematopoietic cells and fibroblasts in mice as well as mesenchymal cells and stromal cells in humans (Huynh et al., 2016; Abugomaa et al., 2020; Yan et al., 2013; Zhu et al., 2015; Shu et al., 2019). CD133, also known as prominin-1, is a five-transmembrane glycoprotein in stem and progenitor cells, which mediate WNT/β-Catenin, Ras/ERK, Src/FAK, PI3K/AKT, Notch, and hedgehog signaling pathways (Kanwal et al., 2018; O'Brien et al., 2007; Huang et al., 2013; Wang et al., 2013; Li D. et al., 2022; Lu et al., 2016).

Epithelial Cell Adhesion Molecule (EpCAM), a homophilic cell-cell adhesion glycoprotein, serves as a prognostic marker, therapeutic target, and anchor molecule on circulating and disseminated tumor cells, which regulates tumor cell adhesion, proliferation, migration, stemness, and epithelial-to-mesenchymal transition (Ni et al., 2013; Leng et al., 2018; Bryan, 2015; Wang et al., 2018c; Alibolandi et al., 2015; Noh et al., 2018; Dai et al., 2017). C-X-C chemokine receptor type 4 (CXCR4, CD184), is involved in WNT/β-Catenin, Notch, hedgehog, PI3K/AKT, JAK/STAT, NF-κB, MAPK, and EGFR/HER2-neu signaling pathways for tumor cell survival, proliferation, migration, and metastasis (El-Benhawy et al., 2021; Serna et al., 2020; Yi et al., 2014; Testa et al., 2018; Xue et al., 2017). Leucine-rich repeat-containing G protein-coupled receptor 5 (LGR5), also known as G protein-coupled receptor 49 (GPR49), belongs to the G protein-coupled receptor family, serves as cell surface-expressed Wnt target gene for cancer stem cell proliferation and self-renewal by regulating Wnt/β-catenin signaling pathway (Takahashi et al., 2011; Green et al., 2019; Nakajima et al., 2016). Aldehyde dehydrogenases (ALDH), consisting of 19 putative members, are involved in tumor cell differentiation, proliferation, invasion, metastasis, and epithelial-mesenchymal transition (Gorodetska et al., 2024; Holah et al., 2017; Namekawa et al., 2020; Louhichi et al., 2018; Gao et al., 2015; Senel et al., 2017). ATP-binding cassette superfamily G member 2 (ABCG2), a cell membrane pump encoded by the *ABCG2* gene, could protect cells against compounds initiating and/or intensifying neoplasia, which is responsible for cancer growth, drug resistance, and recurrence (Wang L. et al., 2020; Xie et al., 2014; Das et al., 2019). Epidermal Growth Factor Receptor (EGFR), activated by receptor overexpression and ligand-dependent/independent mechanisms, serves as an essential receptor tyrosine kinase regulator for cancer stem cell functions, which include stemness, metabolism, immunomodulatory activity, dormancy, and therapy-resistance (Rossini et al., 2020; Mouillet-Richard, 2022; Steelman et al., 2016; Si et al., 2019; Liu Y. et al., 2023).

### Targets and drug discovery

CSC microenvironment is involved in tumor invasion, differentiation, metastasis, angiogenesis, genotoxicity, and self-renewal, which includes vascular niches, hypoxia, extracellular matrix, tumor-associated macrophages, as well as cancer-associated fibroblasts and mesenchymal stem cells (Xue et al., 2015). Plerixafor (AMD3100), the most well-characterized CXCR4-targeted drug, has been applied for the phase I/II study of non-Hodgkin’s lymphoma (NHL), acute myeloid leukemia (AML), and myelodysplastic syndrome (MDS) (Cashen et al., 2008). LY2510924, a potent and selective CXCR4 antagonist, has been investigated for the phase II study of renal cell carcinoma and small-cell lung cancer in the combination of sunitinib and carboplatin/etoposide respectively (Hainsworth et al., 2016). The combined therapy of LY2510924 with other drugs is under clinical trials for gliomas (NCT03746080, NCT01977677, and NCT01288573) and multiple myeloma (NCT00103662, NCT01220375, and NCT00903968) (Yang L. et al., 2020). Understanding the therapeutic targets in CSCs is essential for developing novel therapies. Emerging technologies are revolutionizing our ability to study CSC biology and identify new targets.

**FIGURE 2:**
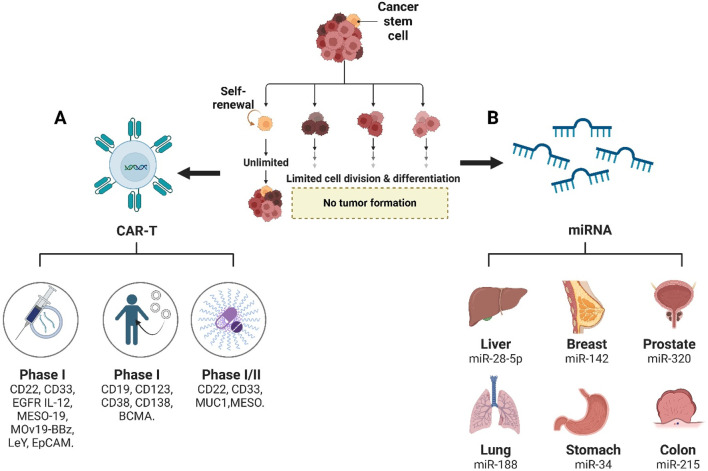
Therapeutic strategies targeting cancer stem cells (CSCs). **(A)** CAR-T cell therapies against CSC surface markers are currently in clinical development, with Phase I trials targeting CD19, CD22, CD33, CD38, CD123, CD138, BCMA, EGFR, IL-12, MESO, and EpCAM, and Phase I/II trials evaluating CD22, MUC1, and MESO. **(B)** Organ-specific miRNA therapeutics for CSC suppression include miR-28-5p in liver cancer, miR-142 in breast cancer, miR-320 in prostate cancer, miR-188 in lung cancer, miR-34a in gastric cancer, and miR-215 in colon cancer.

Chimeric antigen receptor (CAR)-T cells are engineered T cells expressing an artificial receptor specific to tumor associated antigens (TAAs), which could induce the release of cytotoxic cytokines, perforin and granzyme (Benmebarek et al., 2019). CSC-targeted CAR-T cell monotherapy has been applied to many clinical trials, which could be clarified into phase I study (CD22 CAR-T, CD33 CAR-T, EGFR IL-12 CAR-T, MESO-19 CAR-T, MOv19-BBz CAR -T, LeY CAR-T, and EpCAM CAR-T), phase II study (CD19 CAR-T, CD123 CAR-T, CD38 CAR-T, CD138 CAR-T, and BCMA CAR-T), and phase I/II study (CD22 CAR-T, CD33 CAR-T, MUC1 CAR-T/PD-1 KO, and MESO CAR-T) (Yang L. et al., 2020). The combined therapy of CSCs-specific CAR-T cells with chemotherapy and radiotherapy could completely eradicate tumors without recurrence risk (Figure 2A), which has been applied in the treatment of glioma (NKG2D CAR-T cells with regional radiotherapy), ovarian cancer (CD133 CAR-NK92 cells with cisplatin), colorectal cancer (EpCAM CAR-NK92 cells with regorafenib) (Masoumi et al., 2021; Weiss et al., 2018; Klapdor et al., 2017; Zhang et al., 2018).

MicroRNAs (miRNAs), small single-stranded 22 nucleotides length RNA molecules, serves as new promising diagnostic and therapeutic tools for CSCs control (Figure 2B), such as proliferation, migration, invasion, differentiation, apoptosis, differentiation, tumourigenesis, and recurrence, which was awarded jointly to Victor Ambros and Gary Ruvkun by the Nobel Prize in Physiology or Medicine 2024 (Nobel Prize, 2024). miR-28-5p overexpression inhibited self-renewal and tumorigenesis of liver CSCs, which depends on the direct target insulin-like growth factor-1 (IGF-1) (Xia et al., 2019). miR-188 inhibited the biological activity of lung CSCs by targeting midkine (*MDK*) and mediating the Hippo pathway (Yang X. et al., 2020). miR-142-3p overexpression could result in reduced mammosphere formation and irradiation survival, with the decrease of CD44, CD133, ALDH1, Bod1, and BRCA2 in breast CSCs (Troschel et al., 2018). miR-34 is involved in self-renewal/differentiation decision-making of gastric CSCs (Jafari and Abediankenari, 2017). miR-320 could to suppress prostate cancer stem-like properties, such as tumorsphere formation, chemoresistance and tumorigenic abilities (Hsieh et al., 2013). miR-497 overexpression could suppress gemcitabine resistance, migration, invasion, and metastasis of pancreatic CSCs by directly targeting nuclear factor kappa B 1 (NFκB1) (Yu Q. et al., 2022). miR-215 serves as CDX1 effector and BMI1 repressor to regulate self-renewal and multipotency of colorectal CSCs (Jones et al., 2015).

### Mechanisms of drug resistance in CSCs

CSCs exhibit both intrinsic and extrinsic drug resistance mechanisms, allowing them to evade conventional therapies and contribute to tumor recurrence. The intrinsic drug resistance mechanisms make CSCs a major challenge in cancer treatment, which include quiescence, enhanced DNA repair, and adaptive signaling pathways (Choi et al., 2020). The extrinsic drug resistance mechanisms involve interactions between CSCs and their surrounding tumor microenvironment or niche, which include niche-mediated protection, immune evasion, and metabolic adaptation (Najafi et al., 2019).

CSC quiescence is a key survival strategy contributing to tumor recurrence, metastasis, and therapy resistance, which is modulated by intrinsic factors (cell cycle regulation, epigenetic modifications, and metabolic adaptation) and extrinsic factors (hypoxia, stromal interactions, and immune surveillance) (Gulaia et al., 2018). Targeting CSC quiescence requires a multi-pronged approach combining epigenetic, metabolic, and niche-disrupting therapies alongside immunotherapy, which include forcing quiescent CSCs into cell cycle/“Wake-Up and Kill” (dormancy pathway inhibitors, epigenetic modulators, and pro-oxidant therapies), inducing permanent dormancy or senescence (p38 MAPK activators, mTOR inhibitors, and senescence-inducing drugs), applying immune-based approaches (checkpoint inhibitors and CAR-T/NK cells), as well as targeting the Niche (CXCR4 inhibitors and anti-angiogenic therapies) (Chen et al., 2016; Cho et al., 2019). CSCs exhibit upregulation of multiple DNA repair mechanisms compared to non-CSC tumor cells, contributing to their therapy resistance, which include homologous recombination (HR), non-homologous end joining (NHEJ), base excision repair (BER), nucleotide excision repair (NER), and mismatch repair (MMR) (Lu and Zhang, 2022). Targeting the enhanced DNA repair capacity of CSCs through protein inhibitors (PARP inhibitors, ATM/ATR inhibitors, and DNA-PKcs inhibitors), epigenetic modulators (HDAC/DNMT inhibitors), ROS-induced DNA damage amplification, and synthetic lethality strategies holds significant potential to improve cancer treatment outcomes (Skvortsov et al., 2015). The adaptive signaling networks of CSCs underscore their role as a “persister” population in tumors (Thakur and Ray, 2017; Vazquez-Santillan et al., 2015; Chi et al., 2022). The adaptive signaling networks of CSCs underscore the need for multimodal therapies that simultaneously disrupt survival pathways, target the TME, and exploit metabolic dependencies (Mengistu et al., 2024). Advances in single-cell sequencing and CRISPR screening could refine CSC-targeted interventions and offer hope for durable remission.

CSC niche provides physical protection, biochemical signaling, and metabolic support, which enable CSCs to evade conventional therapies (Plaks et al., 2015). The niche-mediated drug resistance mechanism involves multiple intricate interactions, including stromal cell crosstalk, extracellular matrix remodeling, and hypoxia-induced adaptations (Kalavska et al., 2020). While niche-targeted strategies are promising, their success hinges on personalized approaches and overcoming the plasticity of CSCs and their microenvironment, which include niche disruption, stromal targeting, and dual-targeting approaches. The key feature of CSCs is their ability to evade immune surveillance, enabling tumor progression, metastasis, and recurrence (Su et al., 2020). The immune evasion mechanisms employed by CSCs are multifaceted, involving alterations in antigen presentation, immunosuppressive microenvironment modulation, and resistance to immune effector functions (Tsuchiya and Shiota, 2021). Personalized, combinatorial approaches, such as immune checkpoint blockade, CSC-directed vaccines, and CAR-T/NK cell therapy, could pave the way for improving clinical outcomes in refractory cancers. Unlike most cancer cells that rely predominantly on glycolysis (the Warburg effect), CSCs exhibit dynamic metabolic adaptations that allow them to thrive in harsh microenvironments, evade immune surveillance, and resist chemo- and radiotherapy (El-Sahli and Wang, 2020). Their unique metabolic adaptations—such as reliance on glycolysis, oxidative phosphorylation (OXPHOS), or fatty acid oxidation (FAO)—have emerged as promising therapeutic targets (Nimmakayala et al., 2021). Metabolic adaptation therapy aims to disrupt these pathways, selectively eradicating CSCs while sparing normal cells, which include glycolysis inhibitors, OXPHOS targeting, and FAO/glutaminase inhibition.

### Emerging technologies

This section explores the impact of emerging technologies such as single-cell omics and CRISPR screening on our understanding of CSC biology. We will discuss how these tools are revolutionizing cancer research and enabling the development of precision medicine approaches. Since 2020, several emerging technologies, such as single-cell omics and CRISPR screens, have significantly advanced CSC research, enabling deeper understanding of their biology and the development of novel therapeutic strategies.

Single-cell technologies have revolutionized CSC research by enabling high-resolution analysis of cellular heterogeneity, plasticity, and functional states within tumors, which provide unprecedented insights into CSC biology, including their origin, regulatory mechanisms, and role in therapy resistance (Zheng et al., 2018). Single-cell RNA sequencing (scRNA-seq) could identify rare CSC populations within heterogeneous tumors, characterize CSC transcriptional programs at unprecedented resolution, discover novel CSC markers and signaling pathways, and analyze CSC plasticity and transitions between stem and non-stem states (Zheng et al., 2024). Single-cell Assay for Transposase-Accessible Chromatin using sequencing (scATAC-seq), a powerful tool to investigate chromatin accessibility at single-cell resolution, could identify CSC-specific regulatory landscapes, transcription factor networks, and potential therapeutic targets (Liu et al., 2024). Single-cell spatial omics/Multi-omics integration, powerful tools to dissect the heterogeneity, microenvironment interactions, and molecular mechanisms of CSCs at unprecedented resolution, could track CSC subpopulations and their spatial expansion over time, link hypoxia-driven metabolic shifts to CSC survival, as well as identify spatially resolved drug-resistance mechanisms (Wang Y. et al., 2024).

CRISPR-based screens enable genome-wide or targeted interrogation of gene function, facilitating the discovery of novel therapeutic targets and mechanistic insights into CSC biology, which pave the way for targeted therapies aimed at eradicating CSCs, potentially improving cancer treatment outcomes (Li et al., 2023). Pooled CRISPR knockout (KO) screens utilize sgRNA libraries to disrupt gene function genome-wide or in focused sets (Joung et al., 2017). CRISPR interference (CRISPRi) and activation (CRISPRa) modulate gene expression without DNA cleavage (Pandey et al., 2024). *In vivo* CRISPR screens are conducted in animal models to study CSC maintenance and metastasis in a physiological context (Su et al., 2024).

The integration of artificial intelligence (AI) into CSC research has revolutionized the understanding, diagnosis, and treatment of cancer, which enhanced CSC identification by analyzing multi-omics data (genomics, transcriptomics, proteomics, and epigenomics). AI could accelerate the discovery of CSC-targeting drugs by screening large chemical libraries and predicting drug-CSC interactions (Shende and Devlekar, 2021; Ramović Hamzagić et al., 2024a). AI models integrating drug sensitivity data with CSC gene expression profiles could identify potential therapeutic vulnerabilities. Deep learning models, such as convolutional neural networks (CNNs), have been applied to histopathological images to detect CSC-rich regions in tumors, improving diagnostic accuracy (Chen et al., 2023; Aida et al., 2020). AI-powered platforms could analyze patient-derived data (e.g., liquid biopsies, circulating tumor cells) to stratify patients based on CSC prevalence and predict individualized treatment responses. Machine learning models combining clinical, genomic, and imaging data have guided precision oncology decisions, optimizing therapy regimens to eliminate CSCs and prevent relapse (Chambost et al., 2022; Ramović Hamzagić et al., 2024b).

### Organoids and precision medicine

Cancer is a genetic disease with inherent genetic instability that generates abnormal proteins, driven by the accumulation of mutations. In 2024, a bleak milestone for the first time with 2 million daily cancer cases in the United States (US), over 611,000 deaths from cancer are projected for the whole year, while more than 1,600 deaths are projected for each day (Cancer.org, 2024). By 2029, there will be around 2.2 million new cancer cases diagnosed in the United Kingdom (UK), with more than 900,000 cancer deaths (Cancer Research UK, 2024).

**FIGURE 3:**
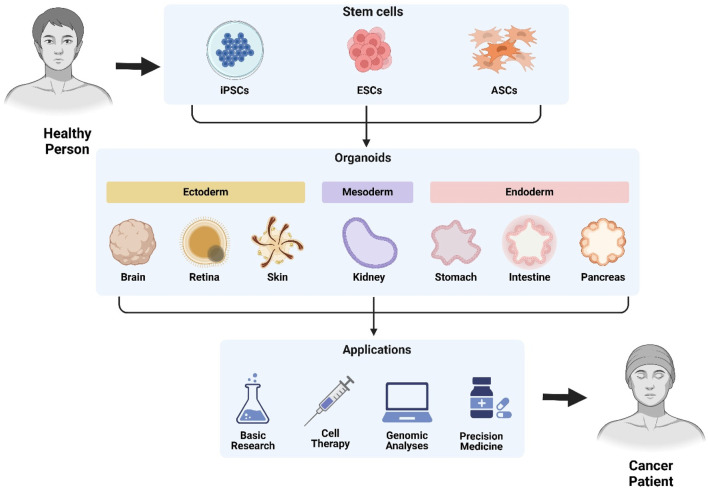
Stem cell-derived organoids: Generation and translational applications. Patient-derived organoids are generated from induced pluripotent stem cells (iPSCs), embryonic stem cells (ESCs), or adult stem cells (ASCs). These differentiate through germ layers (ectoderm, mesoderm, endoderm) into functional organoids including brain, retina, skin, kidney, gastric, intestinal, and pancreatic models. Applications span basic research, cell therapy, genomic analyses, precision medicine, and cancer modeling.

Organoids, three-dimensional (3D) multicellular cultures, are generated from adult stem cells (ASCs) and pluripotent stem cells (PSCs) in many tissues (brain, liver, skin, eye, kidney, lung, stomach, intestine, thyroid, and inner ear), which are widely applied in tissue development, tumorigenesis and cancer therapy due to their capabilities of self-renewal and self-proliferation (Zou et al., 2024). Adult stem cell-derived organoids retain organ identity and genome stability, which could serve as an unlimited source for replacing damaged tissues due to their prospectivities of differentiating into virtually all present organ cellular lineages (Drost and Clevers, 2017). Pluripotent stem cell-derived organoids are established from embryonic stem cells (ESC) and induced PSC (iPSC), which could differentiate into three germ layers (endoderm, mesoderm, and ectoderm) as well as transfect with four transcription factors (Klf4, Oct3/4, Sox2, and c-Myc) (Takahashi et al., 2007). Cancer organoids have been successfully established from both ASCs and PSCs for the maintenance of interpatient and intratumor variation as well as the capture of tumor heterogeneity of individual patients (Figure 3), which are urgently needed for precision medicine, therapy resistance, and cancer progression.

Patient-derived organoids (PDOs) generate ‘living’ organoid biobanks to retain the heterogeneous genetic composition in various cancers, such as breast cancer (Sachs et al., 2018), esophageal adenocarcinoma (Li et al., 2018), gastric cancer (Yan et al., 2018), which is applied in drug efficacy screenings and drug discovery validations. Two complementary strategies have been applied in PDOs for understanding cancer molecular genetics. The first strategy is PDO mutational analysis through whole-genome sequencing (WGS), whole-exome sequencing (WES), and targeted sequencing, which could confirm PDO functions on representing inter-tumor heterogeneity and forming the basis of the inter- and intra-tumor diversification (Michels et al., 2020). The second strategy is probing tumorigenesis mutations through gene editing, which could replicate key features of cancer progression (niche factors independence, chromosome instability, metastasis ability, aneuploidy, and invasiveness), as well as investigate mutations roles in DNA mismatch repair genes (*MLH1*, *BRAF*, *TP53*, and *BAP1*) (Drost et al., 2015; Fessler et al., 2016).

Patient-derived cells (PDCs) are generated from tumor specimens (Li et al., 2025). PDC can reflect patient tumor characteristics and clinical responses, but lack diversity in terms of cell type, spatial organization, and tumor microenvironment. Patient-derived xenograft (PDX) model can effectively replicate tumor growth and preserve tumor heterogeneity, which requires long culture time, high procedure cost, and low transplantation rate (Invrea et al., 2020). The major obstacle of personalized medicine is the lack of effective preclinical models for accurately predicting drug response, sensitivity, and resistance (Voest and Bernards, 2016). Compared with PDCs and PDX, PDOs can better capture and retain the molecular, cellular, genetic, histological, and heterogeneous phenotypes of patient‐specific tumors, which are much more suitable for cancer-personalized therapies to identify key targets and signaling pathways as well as develop incorporate cells of the tumor microenvironment (stromal cells and immune cells). The ovarian cancer PDOs could show accuracy in reflecting clinical responses of patients to platinum-based chemotherapy (Tao et al., 2022). The colorectal cancer PDOs could predict cytoreductive surgery responses followed by hyperthermic intraperitoneal chemotherapy (Ubink et al., 2019). Pancreatic cancer PDOs could accurately predict chemotherapy drug resistance as well as advance personalized medicine (Tiriac et al., 2018). The gastrointestinal cancer PDOs could be used for rapid and actionable drug detection (Gao et al., 2021).

The establishment of PDO biobanks, large-scale collections of organoids from diverse patients, could enable high-throughput drug screening (HTS), which facilitate the identification of novel therapeutics and personalized treatment strategies (Vlachogiannis et al., 2018). PDO biobanks could enable “clinical trial in a dish” approaches by testing drug responses across diverse patient populations, which bridge the gap between preclinical research and clinical application (Herpers et al., 2022). By capturing patient diversity and enabling large-scale pharmacological testing, PDO biobanks hold immense promise for accelerating drug development and advancing precision medicine (Yu YY. et al., 2022; Cui et al., 2024). Future advancements, such as microfluidics, AI-driven image analysis, and organ-on-chip technologies, could enhance the utility of organoid biobanks in drug screening.

While PDOs offer valuable insights into tumor biology and therapeutic responses, it is important to recognize their limitations. Organoids often lack a complete immune component, which can restrict their utility in studying immune responses and the effectiveness of immunotherapies. Additionally, the absence of a vascular system in organoids hinders the accurate modeling of tumor angiogenesis and drug delivery mechanisms. Inter-laboratory variability and challenges in standardizing culture conditions also affect the reproducibility of results, emphasizing the need for improved protocols and quality control measures. Regarding iPSCs, the process of generating these cells through reprogramming somatic cells involves the use of transcription factors delivered by viral vectors, which can lead to insertional mutagenesis and genomic instability. Furthermore, iPSCs may retain epigenetic memory from the original cell type, potentially influencing their differentiation potential and phenotype. Despite these challenges, iPSCs hold significant promise for disease modeling, drug screening, and regenerative medicine, particularly when used to generate organoids that closely resemble patient-specific tissues. To address these limitations, ongoing research is focused on improving organoid models by incorporating immune cells and developing vascularized structures. Additionally, advancements in iPSC reprogramming techniques aim to minimize genomic and epigenetic issues, enhancing the safety and reliability of these cells for clinical applications. By acknowledging and addressing these challenges, we can improve the utility and applicability of organoids and iPSCs in cancer research and personalized medicine.

### From bench to bedside: challenges and opportunities

CSC research is driving precision oncology trials by uncovering vulnerabilities in therapy-resistant populations. Successful translation requires combination strategies, biomarker validation, and innovative trial designs to eradicate CSCs and improve long-term survival. Integrating CSC research into clinical practice offers a paradigm shift from symptom-focused to rhythm-focused medicine, improving outcomes across multiple disciplines.

While preclinical studies have identified promising CSC-targeting approaches, their clinical translation remains difficult due to biological, technical, and practical hurdles. Many CSC-associated pathways are crucial for normal stem cell function, which may cause severe side effects (compensatory mechanisms, gut toxicity, and hematopoietic suppression) by pathway inhibition. Many CSC-targeting agents show efficacy in animal xenograft models but fail in human clinical trials due to CSC biology differences. CSCs exhibit substantial heterogeneity across cancer types and even within individual tumors, which make universal targeting difficult. Non-CSCs can dedifferentiate into CSCs under stress (e.g., chemotherapy or radiation), which lead to therapeutic escape. Available solution could focus on precision medicine approaches to selectively eliminate CSCs while sparing normal stem cells, ultimately improving cancer treatment outcomes, which include epigenetic modulation (DNA methylation and histone modification), drug delivery (nanoparticles), signature identification (single-cell RNA sequencing and liquid biopsies), and long-term outcomes (progression-free survival and recurrence rates).

## Discussion

Our comprehensive review of cancer stem cells (CSCs) in personalized therapy underscores the intricate role these cells play in tumor progression, therapeutic resistance, and metastasis. While our primary focus has been on the molecular pathways and regulatory mechanisms governing CSC behavior, it is essential to broaden our perspective to include the ecological and evolutionary aspects of cancer.

### Variability in CSC markers

The identification and targeting of CSCs rely heavily on specific biomarkers such as CD44 and CD133. However, the expression levels and functional relevance of these markers can vary significantly across different tumor types and even within the same tumor (Zhou et al., 2021; Liu and Wang, 2024). This heterogeneity raises important questions about the robustness of these markers as universal indicators of CSCs. The clinical translation of CSC-targeted therapies must account for this variability to ensure accurate identification and effective targeting of CSC populations. Future research should focus on identifying more reliable and universal CSC markers, possibly through the integration of multi-omics approaches and functional assays.

### Multifactorial nature of therapeutic resistance

CSC-mediated therapeutic resistance is a complex phenomenon involving multiple signaling pathways and cellular mechanisms. Key pathways such as Wnt/β-catenin, Notch, and Hedgehog play pivotal roles in maintaining CSC stemness and mediating resistance to conventional therapies (Steinbichler et al., 2018; Phi et al., 2018). However, the interplay between these pathways and their contribution to resistance is not fully understood. Conflicting evidence regarding the activation status of these pathways in different CSC populations highlights the need for further investigation. Ethical considerations are paramount when manipulating these pathways, as unintended consequences could impact patient safety. Future studies should aim to elucidate the precise mechanisms by which these pathways interact and contribute to therapeutic resistance, paving the way for the development of more effective therapeutic strategies.

### Dynamic interactions within the tumor microenvironment (TME)

The TME is a dynamic and heterogeneous milieu composed of various cell types, including immune cells, fibroblasts, endothelial cells, and the extracellular matrix (ECM). This complex environment plays a crucial role in tumor progression, metastasis, and therapeutic resistance (Bilotta et al., 2022; Wang L. et al., 2024). CSCs do not exist in isolation; they interact extensively with the TME, and these interactions are bidirectional. CSCs can secrete factors that recruit and modulate immune cells, creating an immunosuppressive environment that fosters their survival. Conversely, the TME provides physical and biochemical cues that support CSC self-renewal and maintenance. For instance, hypoxic conditions can enhance CSC stemness, while stromal cells can secrete growth factors and ECM components that promote CSC survival and therapeutic resistance. Exosome-mediated communication is another critical aspect of TME-CSC interactions. CSCs release exosomes containing microRNAs and proteins that can reprogram neighboring cells and modify the TME, further supporting CSC maintenance and therapeutic resistance. Understanding these complex interactions is essential for developing targeted therapies that disrupt the supportive niche of CSCs and sensitize them to conventional treatments.

### Tumor ecosystem and CSC behavior

Viewing the TME as a complex ecosystem that includes immune cells, fibroblasts, and the extracellular matrix provides a broader perspective on CSC behavior. This ecosystem perspective highlights the role of “stemness” as a survival strategy employed by CSCs, allowing them to mimic normal stem cell characteristics to thrive in challenging microenvironments (Luo, 2023; Luo, 2025). Recent research suggests that understanding the tumor ecosystem is crucial for addressing critical clinical issues such as recurrence and metastasis. The tumor ecosystem can be seen as a dynamic system where CSCs, along with other cell types, engage in reciprocal interactions that influence tumor growth and treatment response. This ecological viewpoint emphasizes the importance of considering the tumor as a whole, rather than focusing solely on CSCs. By understanding the complex web of interactions within the tumor ecosystem, researchers can develop strategies that target not only CSCs but also the supportive microenvironment that sustains them. This approach is essential for addressing the challenges of recurrence and metastasis, which are often driven by the adaptive capabilities of the tumor ecosystem. Integrating insights from the tumor ecosystem and ecological pathology allows for a more holistic understanding of CSC behavior and therapy resistance. This broader perspective is crucial for developing effective therapeutic strategies that target both the molecular pathways and the ecological dynamics of the tumor.

### Metabolic adaptations of CSCs

CSCs exhibit distinct metabolic adaptations that support their survival and stemness under stressful conditions (Chen et al., 2021; Masoudi et al., 2024). While lipid metabolism has been a focus, glycolysis and oxidative phosphorylation also play significant roles in CSC metabolism. The Warburg effect, characterized by increased glycolysis even in the presence of oxygen, provides CSCs with rapid energy production and intermediates for biosynthetic pathways. This metabolic shift supports rapid proliferation and biomass production, contributing to CSC survival and therapeutic resistance. Oxidative phosphorylation, although less prominent in CSCs, can be utilized under certain conditions, providing flexibility in energy metabolism. Lipid metabolism, through fatty acid oxidation and lipogenesis, supports membrane biogenesis and signaling, further enhancing CSC maintenance. The integration of these metabolic pathways highlights the metabolic flexibility of CSCs, which must be considered when designing targeted therapies. Inhibiting multiple metabolic pathways simultaneously could enhance the efficacy of therapies by disrupting the metabolic plasticity of CSCs.

### Ethical, safety, and regulatory considerations

The clinical application of advanced experimental strategies, such as CRISPR, CAR-T, and PDOs, raises critical ethical, safety, and regulatory considerations. CRISPR-based therapies involve precise genetic modifications that could have unintended consequences, necessitating rigorous preclinical testing and ethical oversight (Laurent et al., 2024). CAR-T cell therapies, while promising, can elicit severe immune reactions, highlighting the need for careful patient selection and monitoring (Sterner and Sterner, 2021). PDOs, as models for personalized medicine, must be validated to ensure they accurately represent patient tumors and respond appropriately to therapies (Tong et al., 2024). Regulatory frameworks must evolve to address the complexities and challenges associated with these advanced strategies. Ensuring the safety and efficacy of novel therapies while balancing innovation and patient benefit is a delicate task that requires ongoing dialogue among researchers, clinicians, ethicists, and policymakers.

In summary, while our review highlights the potential of CSC-targeted therapies, it is imperative to address the broader ecological and evolutionary aspects of cancer to improve clinical outcomes. Future research should focus on understanding the dynamic interactions within the tumor ecosystem and leveraging this knowledge to develop innovative combination therapies.

## Conclusion and future prospectives

In this review, we summarize valuable insights regarding suitable cancer stem cells (CSCs)-associated signaling pathways, important biomarkers, and therapeutic approaches, which is essential to elucidate CSC functions in tumor metastasis, therapy resistance, and immune escape. Advances in stem cell cultures provide the foundation to powerful three-dimensional organoid technology, which could accurately recapitulate molecular characteristics, genomic alterations, expression profiles, organ structures, specific functions, and tumor microenvironment. The application of patient-derived organoids (PDOs) is beneficial for the comprehensive development of high-throughput drug screening, therapy response, and personalized medicine, which can undoubtedly alleviate serious drug attrition for drug development as well as guide optimized therapeutic strategies for individual patients.

Although CSCs, especially PDOs, show great potential in cancer research, clinical applications, and personalized therapies, there are still many challenges, bottlenecks, and difficulties remaining to be solved in further research. Firstly, establish standardized and optimized organoid culture conditions for improving large-scale reproducibility, reducing culture time, reserving genetic information, and avoiding new mutations. Secondly, build a PDO co-culturing system with immune cells and fibroblasts to replicate and mimic patient-specific immune environments among different tumor types and subject groups. Thirdly, apply genetic manipulation tools in organoid testing platforms to facilitate high-throughput drug screening, clinical practice, therapy guidance, and regenerative medicine.

By reshaping the narrative flow and providing a clear structure, this review aims to deepen the reader’s understanding of CSC biology and its implications for personalized cancer therapy.
